# Potential of termite mounds and its surrounding soils as soil amendments in smallholder farms in central Uganda

**DOI:** 10.1186/s13104-020-05236-6

**Published:** 2020-08-27

**Authors:** Samuel Obeng Apori, Marius Murongo, Emmanuel Hanyabui, Kofi Atiah, John Byalebeka

**Affiliations:** 1 African Center of Excellence in Agroecology and Livelihood System, Faculty of Agriculture, Uganda Martyrs University, Nkozi, Uganda; 2 Department of Soil Science, University of Cape Coast, Cape Coast, Ghana; 3 Faculty of Agriculture, Uganda Martyrs University, Nkozi, Uganda; 4 Ecological organic agriculture initiative, Nairobi, Kenya

**Keywords:** Nutrient index, Surrounding soil, Chemical properties

## Abstract

**Objectives:**

The low fertility of highly weathered soils has been a major problem for resource-constrained smallholder farmers. In central Uganda, smallholder farmers have been collecting termite mound soils anywhere around the termite mound to improve their soil fertility. However, no studies have been conducted on which sections of the termite mounds consist of high soil nutrients. This study was conducted to assess selected major soil essential plant nutrients of soils collected from the top of the mound (TPMS), and the basal part of the mound (BPMS). The surrounding soil samples were collected from five, fifteen, and thirty meters away from the mound (TMSS1, TMSS2, and TMSS3 respectively), covering ten termite mounds in five different maize fields in central Uganda.

**Results:**

TPMS and BPMS had significant (P-value < 0.05) higher N, P, K, OC, Ca and Mg levels than TMSS1, TMSS2, and TMSS3. However, OC levels in BPMS was higher than TPMS. On the whole, termite mounds are beneficial as a source for essential plant nutrients. It will be best if smallholder farmers could collect the termite mound soils from the top and the basal part of the mound to improve the fertility of their soil.

## Introduction

In the tropical and subtropical Agroecosystem, the destructions and damages to crops and farm structures by termites have resulted in the reduction of crop productivity [1, 2]. Out of the estimated total number of 2600 described species of termites, few have been considered as a major pest of food crops such as cereals, roots, tubers, legumes and fruit trees [3–6]. Despite being considered as pests, termites are biological indicators of soil fertility and ecosystem engineers [7–11]. Termite’s activities such as collection and transportation of living and dead plants, animal materials, soil particles, and burrowing lead to the improvement of soil physicochemical properties and microbial population and diversity of the termite mound and their surrounding soils [12–17]. In central Uganda, most large termites mounds are built by *Macrotermes subhyalinus* and *Macrotermes bellicosus* and termite mounds found on farmlands are estimated to be on average 10–15 mounds per acre of land of a density between 0.1 and 3.4 per acre [18].

Soils found in central Uganda, are highly weathered *Acrisol* and *Ferrasols* [19] and are typically characterized by strong acidity, low cation exchange capacity, low nutrient retention capacity, and low available phosphorus [20, 21]. The low fertility of the weathered soils has been a major problem for smallholder farmers who have limited financial resources to purchase commercially available fertilizers. To solve the problems created by these soils, poor smallholder farmers have been collecting soils from the termite mounds to amend their poor soils either solely or in combination with organic resources and fertilizers [22].

To date, there has not been any systematic study done in this sector of Uganda to ascertain which sections of the termite mounds consist of higher levels of macroelements to better inform smallholder farmers on which part should be harvested. The current practice amongst these farmers has been the collection of the surrounding soil from these termite mounds due to ease of collection compared to collection from other sections of the termite mounds. Due to the unavailability of or paucity of information regarding these practices amongst resource-poor farmers in central Uganda necessitated this study. Therefore, the objective of this study was to assess the soil macronutrients, reactivity (pH) and organic carbon contents of the different sections of the termite mounds and their surrounding soils. The main hypothesis being tested is that termite mounds and their surrounding soils differ in the nutrients and soil quality parameters being assessed. The results obtained from this study will improve the knowledge and practice of current integrated soil fertility management (ISFM) of resource-poor smallholder farmers in central Uganda.

## Main texts

### Methods: study area

The study was conducted in a maize field in Nkozi sub-county. Nkozi was chosen due to its widespread high termite mounds density. This study area lies at the equator with coordinates 0.0023° N, 32. 0139° E. The area receives a bimodal rainfall pattern with a mean annual rainfall of 1100 mm with minimum annual temperature ranges from 12 to 23° C and the maximum from 23 to 36° C, respectively.

#### Soil sampling

**Fig. 1:**
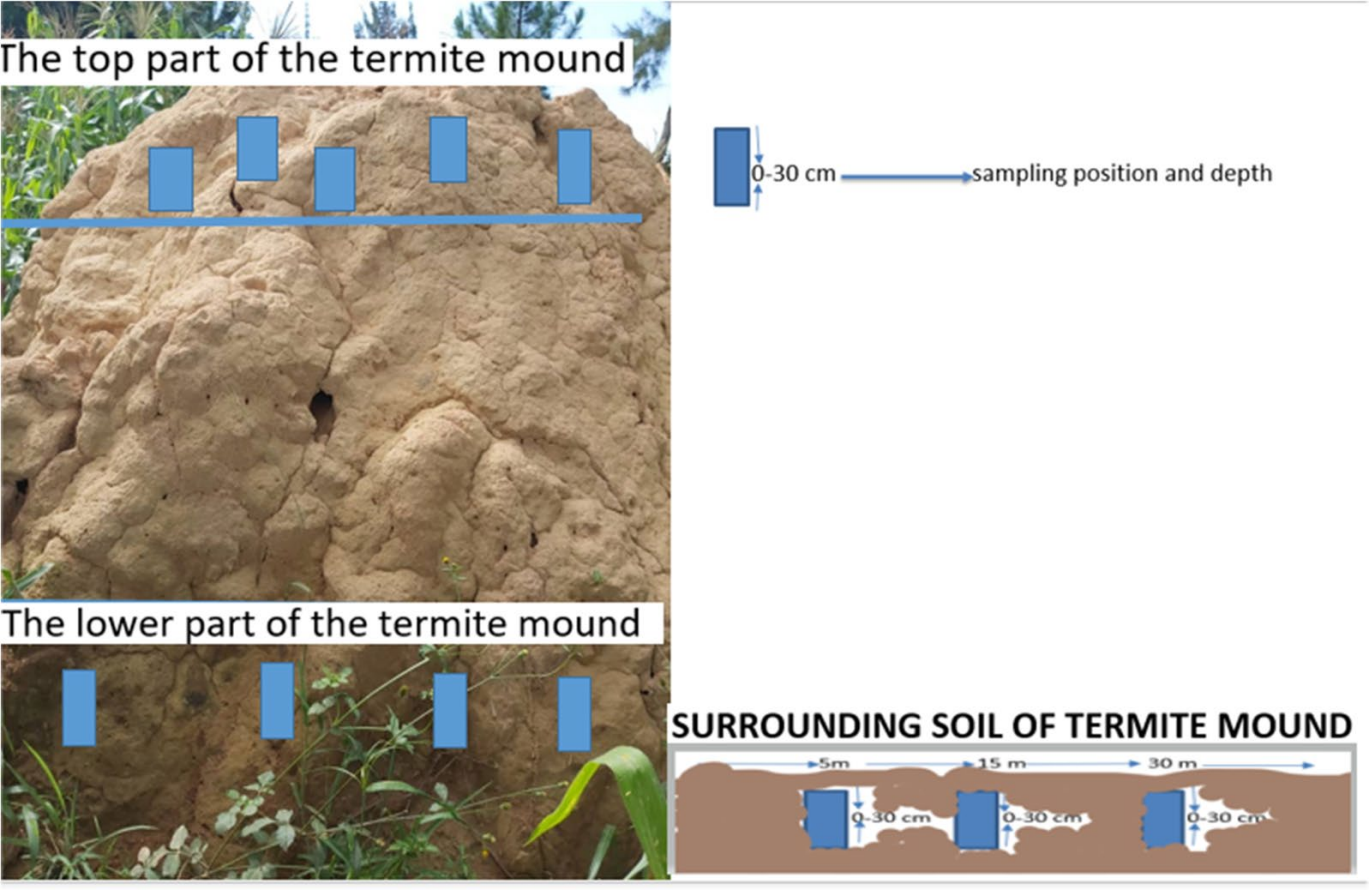
Sampled sections of a termite mound (top and base), the surrounding soil at three distances and a sample depth of 0–30 cm.

Random soil sampling was done on ten termite mounds above three meters height found in five different maize fields. Two termite mounds were sampled from each of the five maize fields. The termite mounds that were mainly occupied by *Macrotermes subhyalinus* and *Macrotermes bellicosus* species were sampled for the current study [18, 23]. Ten termite mounds were used as a sampling points. Soil samples were taken at the depth of 30 cm on the top of the mound (TPMS), and the basal part of the mound (BPMS). The surrounding soil samples were collected from five, fifteen, and thirty meters away from the mound (TMSS1, TMSS2, and TMSS3, respectively) using a soil auger (Fig. 1). The distances selected for the soil sampling of the surrounding soils were chosen to determine the variation of soil nutrients as the distance of surrounding soil increases [24]. The ten termite mounds were selected on a uniform slope in the five maize fields of average size of 4.5 acres. Five composite soil samples were taken from each of the ten mounds and their surrounding soil. The soil samples were air-dried for two weeks after sampling from the field, sieved through a 2 mm mesh sieve and packed into sample bags and kept for soil analysis.

#### Physicochemical analysis of the soil samples

The soil pH was determined in soil: water suspension (1:5) [25]. The total organic carbon was determined by the colorimetric method [26]. Available P was analyzed using the bray 1 acid method [27]. Total nitrogen (N) was determined by the Kjeldahl method [28]. K was determined by a flame photometer [29] whiles Ca^2+^ and Mg^2+^ were determined by the atomic absorption spectrophotometer after extracting with 1.0 M neutral ammonium acetate [29].

#### Nutrient availability indexation

To evaluate the nutrient status of the soil in the study area, pH, organic carbon, available P, exchangeable K, calcium, magnesium and total nitrogen were calculated based on the specific rating chart (Additional file 1: Table S1) The nutrient index in soils were calculated using the method adopted by [30] (Additional file 1: Table S2): Nutrient index (N.I.) = (L × 1 + NM × 2 + NH × 3) / TNS**,** Where L = Number of samples in the low category; M = Number of the sample in the medium category; H = Number of the sample in High category, TNS = Total number of samples. The nutrient index is used to predict the sufficiency of each soil quality indicators in fertile soil using soil test results obtained from the laboratory.

#### Data analysis

The data was analyzed using GenStat Edition 12 statistical software. One way ANOVA was used to compare some selected soil chemical properties of a termite mound and surrounding soil. Means were separated using Fisher’s Unprotected Lsd at 0.05 significance level.

### Results

#### Soil fertility assessment of selected properties of soil samples from termite mounds and their surrounding soil

Soil samples TPMS and BPMS had significantly (P-value < 0.05) higher contents of organic carbon, N, P, K, and Ca levels compared to samples TMSS1, TMSS2, and TMSS3. There was no significant (P-value > 0.05) difference in pH among the soil samples. The pH ranged from 5.72 to 6.18. Organic carbon contents were significantly (P-value < 0.05) higher in the top (TPMS) than any other section of the mounds plus its surrounding soils (Table 1). Organic carbon ranged from 1.59 to 0.47%. However, no difference exists between TMSS2 and TMSS3. TMSS2 and TMSS3 which recorded OC of 0.57 and 0.47%, respectively. The total N was significantly (P-value < 0.05) different from samples across the termite mounds sections. The values of total N across all samples ranged from 0.15 to 0.05% with the highest and the least being recorded by TPMS and TMSS3, respectively. There was significantly (P-value < 0.05) higher contents of exchangeable K amongst samples of TPMS and BPMS compared to the surrounding soils. However, there was no significant (P-value > 0.05) difference between TMSS2 and TMSS3. TMSS2 and TMSS3 recorded a potassium level of 0.18 and 0.15 cmol kg^−1^, respectively. The available phosphorus was higher in TMSS1 than TMSS2 and TMSS3. Available phosphorus for sample TMSS1 recorded 7.85 mg kg^−1^ while TMSS2 and TMSS3 recorded available phosphorus of 4.61 and 3.38 mg kg^−1^, respectively. Exchangeable bases (Mg, Ca) were significantly higher in TPMS and BPMS as compared to TMSS1, TMSS2 and TMSS3. However, calcium was higher in TMSS1 and TMSS2 than TMSS3. TMSS1 recorded a calcium level of 4.51 cmol kg^−1^ while TMSS2 and TMSS3 recorded 4.03 and 3.51 cmol kg^−1^, respectively (Table 1).

**Table 1:** Selected chemical properties of soil samples of termite mounds and their surrounding soil

Soil samples	pH (H_2_O)	O.C (%)	N (%)	Mg (cmol kg^−1^)	Ca (cmol kg^−1^)	K (cmol kg^−1^)	Av. P (mg kg^−1^)
TPMS	5.72a	1.59d	0.15c	3.79c	9.13e	0.32c	16.47d
BPMS	5.71a	1.39c	0.15c	3.86c	8.40d	0.31c	15.64d
TMSS1	5.99ab	0.80b	0.07b	3.24b	4.51c	0.26b	7.85c
TMSS2	5.68a	0.57a	0.08b	3.43bc	4.03b	0.18a	4.61b
TMSS3	6.18b	0.47a	0.05a	2.27a	3.51a	0.15a	3.38a
P-value	NS	***	***	***	***	***	**
SED	0.18	0.05	0.01	0.23	0.21	0.02	0.55

Means followed by the same letter in each column are not significantly different at P ≤ 0.0 5 using Fisher’s unprotected LSD.

*NS*  not significant

*significant at P < 0.05

**significant at P < 0.01

***significant at P < 0.001

#### Nutrient availability index of termite mounds and surrounding soil

Soil samples from TPMS, BPMS and TMSS1 recorded a high carbon content, TMSS2 recorded moderate and TSS3 recorded a low level of carbon content. Also, the nutrient index result indicated a high level of nitrogen content in soil samples TPMS and BPMS while TMSS1, TMSS2 and TMSS3 indicated low nitrogen contents. All the soil samples recorded a moderate level of magnesium content. TPMS, BPMS, TMSS1 recorded a high level of calcium content, TMSS2 for moderate and TMSS3 recorded low calcium content. Soil samples from TPMS and BPMS recorded a moderate level of Available phosphorus while TMSS1, TMSS2 and TMSS3 recorded low content. Potassium content was also high in TPMS and BPMS while TMSS1, TMSS2 and TMSS3 recorded a moderate level of potassium (Table 2).

**Table 2:** Nutrient index of selected properties of 10 soil samples of termite mounds and its surrounding soil

Soil sample locations	O.C (%)	N (%)	Mg (cmol kg^−1^)	Ca (cmol kg^−1^)	K (cmol kg^−1^)	Av. P (mg kg^−1^)
TPMS						
Nutrient index	3	3	2.1	3	3	2
Remarks	High	High	Medium	High	High	Medium
BPMS						
Nutrient index	3	2.4	2	3	3	1.7
Remarks	High	High	Medium	High	High	Medium
TMSS1						
Nutrient index	2.8	1.6	2	2.6	2.3	1
Remarks	High	Low	Medium	High	Medium	Low
TMSS2						
Nutrient index	1.8	1.2	2	2	2.2	1
Remarks	Medium	Low	Medium	Medium	Medium	Low
TMSS3						
Nutrient index	1.6	1	1.9	2	2.1	1
Remarks	Low	Low	Medium	Medium	Medium	Low

### Discussion

#### Soil nutrients and soil quality indicators dynamics of the termite mound

The pH of the soil from the termite mounds and its surroundings was weakly acidic. Li et al. [31], stated that most termite mounds are in acidic and weakly alkaline soils since higher soil pH leads to termite inactivation. Weak acidic soils play a significant role in increasing soil phosphorus availability [32], improve nutrient retention capacity [33] and creating favorable environmental conditions for soil microorganisms responsible for nitrogen and carbon cycling [34]. Most pH requirement for crops ranges from 5.5–5.8, therefore, collection of termite mound soils for amendments could meet most of the pH requirement for crops [35, 36]. The higher organic carbon percentage in the termite mound was due to the organic materials used in its construction, and the types of food they eat. Termites feed on plant materials (live and dead plants, litter in various stages of decay), dung, soil and specialized food such as lichens [2, 37]. Some of the termites die and decay, thereby contributing to increasing soil organic carbon. The results obtained were similar to several other studies which concluded that organic carbon is higher in termites mound than the surrounding soil [22, 28, 38, 39]. The OC recorded by the termite mounds and their surrounding soil falls above the SOC critical threshold at 0.4%. However, collecting soil TPMS for application on the field will results in high crop productivity than the other sections of the mound [40].

The higher amount of N, P and K in termite mound compared to surrounding soils was due to the cumulative effect of organic matter by the termites in their mound. The accumulation of the organic matter in the termite mound increases plant macronutrients such as nitrogen, phosphorus and potassium. De BRUYN and Conacher [41], stated that the capacity of termites to increase nutrient levels such as nitrogen, phosphorus potassium, calcium and magnesium are dependent on the rate of organic material incorporation and the type of artificial mound made by the termites. In the work of Arshad [37], where termite mounds were combined with soil, the results showed high percentages of plant nutrients such as calcium, mineral nitrogen, extractable potassium and available phosphorus compared to the control. Jouquet et al*.* [42], stated that the grinding of soil particles by termite mandibles in the saliva-rich environment of the buccal cavity increases the surface area exposed to the surrounding, solution and then releases interlayer K and adsorption of hydrated or polar ions between the layers. Calcium content and magnesium were higher in the mound than the surrounding soil even though the nutrient index determination showed that there is a moderate level of calcium content for both the various section of the mound and their surrounding soils. The results are in agreement with Chisanga et al. [22] who reported a high concentration of Ca in soil from the top part of the termite mound.

### Conclusion and recommendation

Both the top and the basal part of termite mound soils are beneficial as a source for major essential plant nutrients compared to the surrounding soil. However, extra research work on amending degraded soils with termite mounds’ soils on plant nutrient availability should be done to elucidate their mechanism of improving soil fertility.

## Limitation

In this study, some soil quality indicator parameters were not determined yet termite activities affect the biological and physical properties of the soil.

## Supplementary information

**Additional file 1: Table S1.** Rating chart for soil parameters and their nutrient indices.** Table S2.** The nutrient Index with range and remark according to Ravikumar andSomashekar [30] was used in the study.

## Data Availability

All data generated or analyzed during this study are included in this manuscript.

## Abbreviations

TPMS: Soil samples from the top part of the termite mound
BPMS: Soil samples from the basal part of the termite mound
TMSS1: Five meters away from the termite mound
TMSS2: Fifteens meters away from the termite mound
TMSS3: Soil samples from thirty meters away from the termite mound
O.C: Organic carbon
Tot. N: Total nitrogen
Av. P: Available P
Ca: Calcium
K: Potassium
Mg: Magnesium
