# Supplementary material for: Potential of termite mounds and its surrounding soils as soil amendments in smallholder farms in central Uganda
Source: BMC Res Notes. 2020 Aug 27;13:397. doi: 10.1186/s13104-020-05236-6 (PMC7457291; doi:10.1186/s13104-020-05236-6)
Supplement: Supplementary file 1 — Additional file 1: Table S1. Rating chart for soil parameters and their nutrient indices. Table S2. The nutrient Index with range and remark according to Ravikumar andSomashekar [30] was used in the study. [file 13104_2020_5236_MOESM1_ESM.docx]

**Table S1. Rating chart for soil parameters and their nutrient indices**

| **Soil pH** | | | |
| --- | --- | --- | --- |
| Range  Soil reaction index | Acidity Neutrality Alkaline  Below 6.0 6.0 -8.0 Above 8.0  I II III | | |
| **Organic matter (OC)** | | | |
| Range (%)  Nutrient index | Low Medium High  Below 4 4-20 Above 20  I II III | | |
| **Total nitrogen** | | | |
| Range (%)  Nutrient index | Low  Below 0.08  I | Medium  0.08-0.15  II | High  Above 0.25  III |
| **Calcium (Ca)** | | | |
| Range ( cmol kg^-1^)  Nutrient index | Low Medium High  Below 1.5 1.5-4.5 Above 4.5  I II III | | |
| **Magnesium (Mg)** | | | |
| Range ( cmol kg^-1^)  Nutrient index | Low Medium High  Below 1.5 1.5-4.5 Above 4.5  I II III | | |
| **Potassium (k)** | | | |
| Range ( cmol kg^-1^)  Nutrient index | Low Medium High  Below 0.08 0.08-0.15 Above 0.25  I II III | | |
| **Available phosphorus ( p)** | | | |
| Range ( mg kg^-1^)  Nutrient index | Low Medium High  Below 15 15-50 Above 50  I II III | | |

| S.N. | Nutrient Index | Value |
| --- | --- | --- |
| III | High | Above 2.33 |
| II | Medium | 1.67-2.33 |
| I | Low | Below 1.67 |

**Table S2. The nutrient Index with range and remark according to Ravikumar and**

**Somashekar [30] was used** in the study
